# Effects of feeding on the physiological performance of the stony coral *Pocillopora acuta*

**DOI:** 10.1038/s41598-020-76451-1

**Published:** 2020-11-17

**Authors:** Yan-Leng Huang, Anderson B. Mayfield, Tung-Yung Fan

**Affiliations:** 1 Institute of Marine Biology, National Dong Hwa University, Pingtung, 944 Taiwan, ROC; 2 National Museum of Marine Biology and Aquarium, Pingtung, 944 Taiwan, ROC; 3 Atlantic Oceanographic and Meteorological Laboratory, National Oceanic and Atmospheric Administration, Miami, FL 33149 USA; 4 Cooperative Institute for Marine and Atmospheric Studies, University of Miami, Miami, FL 33149 USA

**Keywords:** Physiology, Zoology

## Abstract

Reef-building corals rely on both heterotrophy and endosymbiotic dinoflagellate autotrophy to meet their metabolic needs. Those looking to culture these organisms for scientific or industrial purposes must therefore consider both feeding regimes and the light environment. Herein the effects of three photosynthetically active radiation (PAR) levels were assessed in fed and unfed specimens of the model coral *Pocillopora acuta* that were cultured in a recirculating aquaculture system (RAS). Half of the corals were fed *Artemia* sp. brine shrimp in a separate feeding tank to prevent biofouling, and fragments were exposed to PAR levels of 105, 157, or 250 μmol quanta m^−2^ s^−1^ over a 12-h period each day. All cultured corals survived the 140-day treatment, and the physiological response variables assessed-buoyant weight, specific growth rate, linear extension, color, and Fv/Fm-were significantly influenced by feeding, and, to a lesser extent, light. Specifically, fed corals grew faster and larger, and presented darker pigmentation; corals fed at the highest light levels grew at the fastest rate (6 cm year^−1^ or 175 mg g^−1^ week^−1^). Given the high physiological performance observed, we advocate the active feeding of brine shrimp in RAS by those looking to cultivate *P. acuta*, and likely other corals, over long-term timescales.

## Introduction

Earth’s coral reefs are in rapid decline due to ocean warming, acidification, intensified typhoons, pollution, overfishing, and other factors. Reef ecosystems have become so marginalized that ex situ coral husbandry and propagation have become increasing important for not only advancing scientific research, but also biobanking and biopreservation^1,2^. Fortuitously, corals have been cultivated for decades for both research purposes and the aquarium trade^3–7^, and recent projects have sought to rear corals for later restoration and out-planting^5,6,8^. Most research corals are cultured in flow-through systems (FTS) featuring natural seawater^9^. However, the recirculating aquaculture systems (RAS) are gaining more widespread utility since, unlike FTS, seawater quality can be better controlled within them^7^. In fact, seawater quality can be modulated to enhance growth and color of corals^1^. Environmental factors such as light, temperature, and water motion, can be programmed to mimic in situ conditions through microprocessor technology, and researchers have successfully simulated conditions that elicit coral spawning ex situ^10^. Co-culture with “live” rocks^11,12^ and heterotrophic feeding^13–21^ have both been shown to enhance coral growth.

Reef corals are mixotrophic and so are dependent on both heterotrophy and the autotrophy of the endosymbiotic dinoflagellates (family Symbiodiniaceae) residing within their gastrodermal cells for nourishment^5,22^. Therefore, it is unsurprising that strong light effects on physiology have been documented in cultured corals^3,4,23,24^. Heterotrophy becomes relatively more important in bleached corals that have lost the capacity for autotrophy, and active feeding has been shown to promote bleaching resilience^3,20,25–28^ and raise coral protein levels, chlorophyll concentrations, photosynthetic rates, and growth rates^16,17,29,30^. However, feeding in RAS usually leads to eutrophication and can ultimately actually thwart coral growth when algal blooms occur within the culture tanks^31^. Using physically separated feeding tanks has addressed this eutrophication issue and increase coral feeding efficiency^32^.

Corals are generally thought to be autotrophic during the day and heterotrophic at night, but the relative importance of light vs. feeding regime is highly variable across species and environmental gradients^18^. For aquaculturists, both light and feeding regime must be optimized^4^. To optimize the aquarium culture of the common Indo-Pacific reef coral *Pocillopora acuta* (formerly synonymized with *P. damicornis*^33^), which is abundant on coral reefs in Taiwan^34,35^, we investigated the effects of both light intensity and heterotrophic feeding (in a separate feeding tank) on its physiology within RAS. *P. acuta* growth has been investigated^9^, and feeding with *Artemia* nauplii has been shown to successfully replace their natural diet^19^. We hypothesized that fed corals would outperform starved ones over a 5-month culture duration.

## Results

### Seawater quality

Seawater quality was generally similar among culture tanks (Table 1), and levels of ammonia, nitrite, nitrate, and phosphate remained below detectable levels (< 0.2 mg L^−1^) during the entire experimental period. However, detectable concentrations of nitrate were documented in the feeding tank, and nitrate concentrations were significantly higher in the feeding tank vs. the culture tanks (Kruskal–Wallis 1-way ANOVA, *p* < 0.001). Please note that alphas of 0.01 and 0.05 were set for all main effect models and post-hoc tests, respectively (described in details in the “Methods”).

**Table 1:** Comparison of seawater chemistry parameters across tanks.

Parameter	Temp.*	Salinity	pH*^a^	Ca^2+a^	Mg^2+^*^a^	KH^a^
Units	°C			mg L^−1^	mg L^−1^	dKH
Culture tank 1	27.4 ± 0.4^A^	34.4 ± 0.5	8.32 ± 0.03^AB^	430 ± 19	1320 ± 28^A^	7.59 ± 0.55
Culture tank 2	27.1 ± 0.6^AB^	34.7 ± 0.5	8.27 ± 0.06^B^	420 ± 14	1302 ± 26^AB^	7.53 ± 0.50
Culture tank 3	27.1 ± 0.1^AB^	34.6 ± 0.5	8.34 ± 0.03^A^	425 ± 15	1284 ± 38^B^	7.58 ± 0.56
Feeding tank	26.9 ± 0.6^B^	34.5 ± 0.5	8.16 ± 0.04^C^	430 ± 16	1275 ± 36^B^	7.69 ± 0.52

Significant differences among tanks were documented for parameters denoted by asterisks (*), and Tukey’s (parametric) or Dunn’s (non-parametric) post-hoc differences (*p* < 0.05) are marked with capital letters. Error bars represent standard deviation. Concentrations of nitrite, nitrate, phosphate, and ammonia were below detectable levels except for nitrate in the feeding tank (5 ± 1 mg L^−1^). *Temp.* temperature.

^a^Data analyzed by non-parametric ANOVA.

### Buoyant weight (BW), specific growth rate (SGR), & total linear extension (TLE)

**Figure 1:**
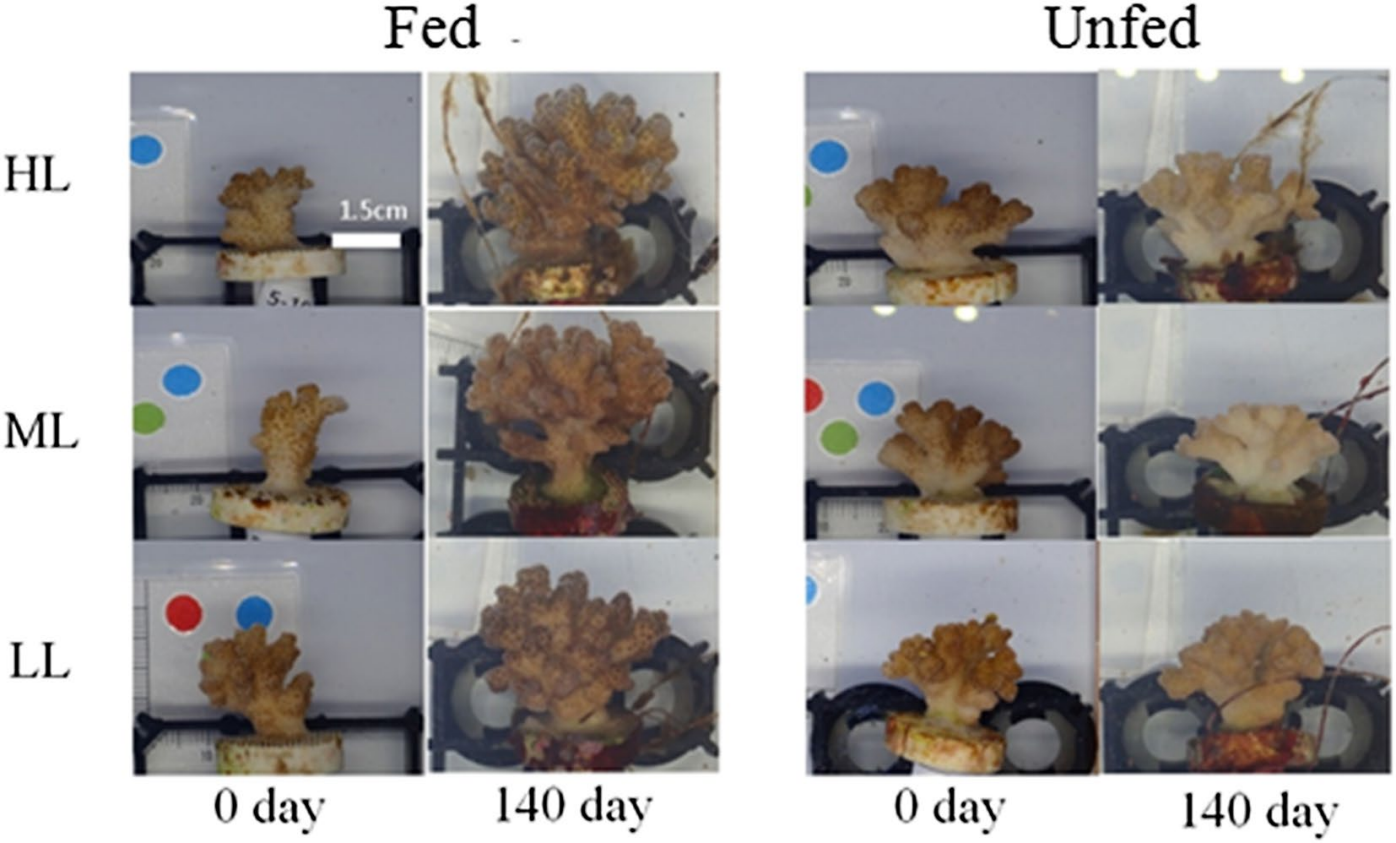
Representative images of corals at the beginning (0-day) and end (140-day) of the experiment. The scale bar applies to all images. *HL* high light, *ML* medium light, *LL* low light.

**Figure 2:**
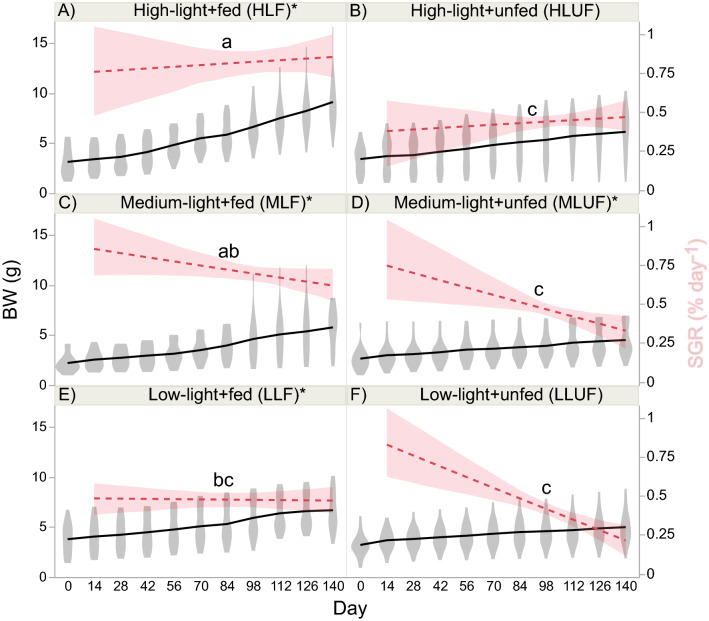
Coral growth. Growth was plotted over time as buoyant weight (BW; grey violin plots with black solid line traversing means at each time; left y-axis), with the specific growth rate (SGR) calculated from day 14 onwards (right y-axis) plotted as a horizontal, hatched, red line bounded by 95% confidence intervals. Lowercase letters above the SGR bands reflect post-hoc SGR differences (*p* < 0.05) across the six light × feeding regime interaction groups. Please note that, although this effect was not statistically significant in the full, mixed-model, repeated measures ANOVA (Table 2), a less conservative, one-way ANOVA across the six groups (box–cox-transformed data) revealed significant differences (*F* = 9.90, *p* < 0.001). When the raw BW changed significantly over time (*p* < 0.01), an asterisk (*) has been placed next to the interaction group abbreviation (e.g., “HLF”) in the legend title.

All 90 corals survived the 140-day experiment (Fig. 1), and BW, which was similar across tanks and treatment groups at day-0 (both *p* > 0.01), generally increased over time (Fig. 2 and Table 2): day-140 > day-0 (pooled across treatments). When considering the raw BW data alone (Fig. 2-left y-axis), there was a time × light × feeding regime effect (Table 2); this is evidenced in Fig. 2 by the observation that BW rose significantly in four of the six treatment groups. The BW of the high-light + unfed (HLUF; Fig. 2B) and low-light + unfed (LLUF) corals (Fig. 2F) did not actually increase significantly over time, though the respective *p*-values were between 0.01 and 0.05. Raw BW was significantly affected by light and was highest at the medium light level (Table 2).

**Table 2:** Repeated measures ANOVA.

Parameter (unit; AICc)				
Source of variation	df	*F*	*p*	Select post-hoc test results
**BW-raw (g; AICc = − 877) (Fig. ****2****-left y-axis)**				
Light	2	6.007	0.004	Medium < others
Food	1	1.725	0.193	
Light × food	2	0.632	0.535	
Time	1	2188	< .0001	Day-140 > day-0
Light × time	2	57.48	< .0001	
Food × time	1	169.4	< .0001	
Light × food × time	2	5.793	0.0032	
Tank[light × food]	12	1.120	0.358	
**SGR (% day**^**−1**^**; AICc = 84) (Fig. ****2****-right y-axis)**				
Light	2	11.022	0.0236	
Food	1	33.73	< .0001	Fed > unfed
Light × food	2	2.077	0.1325	
Tank	2	3.810	0.1185	
**Linear extension (cm; AICc = − 215) (Fig. ****3****-left y-axis)**				
Light	2	4.624	0.0129	
Food	1	4.145	0.0454	
Light × food	2	0.786	0.4594	
Time	3	263.7	< .0001	120 > 30
Light × time	6	3.674	0.0021	
Food × time	3	22.15	< .0001	Fed-120 > unfed-120
Light × food × time	6	2.634	0.0190	
Tank[light × food]	12	0.698	0.749	
**Linear extension**^**a**^** (% increase; AICc = 395) (Fig. ****3****-right y-axis)**				
Light	2	10.31	0.0264	
Food	1	41.30	< .0001	Fed > unfed
Light × food	2	3.052	0.0529	
Tank	2	0.493	0.6434	
**F**_**v**_**/F**_**m**_** (unitless; AICc = − 3831)(Fig. ****4****)**				
Light	2	4.048	0.0218	
Food	1	24.82	< .0001	Fed > unfed
Light × food	2	2.924	0.0605	
Time	1	126.1	< .0001	
Light × time	2	12.38	< .0001	
Food × time	1	32.12	< .0001	
Light × food × time	2	1.807	0.1656	
Tank[light × food]	12	16.72	< .0001	Culture tank 1 > others
**Color**^**b**^** (raw scores; AICc = 12,821) (Fig. ****5****-left y-axis)**				
Light	2	61.59	< .0001	Low > high
Food	1	340.5	< .0001	Fed > unfed
Light × food	2	6.779	0.0021	Low-unfed > high-unfed
Time	3	212.9	< .0001	
Light × time	6	7.721	0.0001	
Food × time	3	430.2	< .0001	
Light × food × time	6	3.600	0.0283	
Tank[light × food]	12	2.194	0.0220	
**Color change**^**b**^** (unitless; AICc = 755) (Fig. ****5****-right y-axis)**				
Light	2	19.42	0.0087	Low > high
Food	1	172.0	< .0001	Fed > unfed
Light × food	2	2.773	0.0690	
Tank	4	0.501	0.640	

For buoyant weight (BW), Fv/Fm, linear extension (raw cm), and color (raw scores), repeated measures ANOVAs of light (three levels) vs. feeding (fed or unfed) over time (140 days with biweekly sampling) were carried out under an unstructured design (i.e., all possible covariances considered). For the specific growth rate (SGR), linear extension (% increase), and color change (final–initial), 2-way ANOVAs (light × feeding regime) were instead conducted with colony as a random effect. All data were box–cox-transformed prior to the mixed model analysis unless stated otherwise, and select post-hoc test results (non-exclusive) have been shown; all others can be found in the respective figures. *AICc* Akaike’s information criterion (penalized for number of parameters).

^a^Untransformed.

^b^Non-parametric repeated-measures ANOVA.

Since corals began the experiment similarly sized, the specific growth rate (SGR) is a better means of assessing coral growth and will hereafter take precedence over BW in describing changes in fragment size. SGR was significantly affected by feeding regime (fed > unfed; Table 2), and, though there was no interaction of light and feeding regime in the repeated measures (RM) ANOVA (Table 2), clear differences among the SGRs of the six interaction groups can be seen in Fig. 2 (right y-axes). Therefore, a less conservative 1-way ANOVA across the six treatment groups was carried out, with the results of post-hoc analyses shown in Fig. 2. SGR was highest in the high-light + fed (HLF) treatment (Fig. 2A; ~ 0.8% day^−1^) and lowest in the LLUF one (Fig. 2F; ~ 0.3% day^−1^). In the former, this equated to a mean quadrupling in size (~ 2 to 9 g; Fig. 2A-left y-axis) over the duration of the 140-day experiment (~ 350 mg week^−1^ increase or ~ 175 mg g^−1^ week^−1^). The HLF group SGR was significantly higher than that of all other treatments except the medium-light + fed (MLF) one, and the mean SGR of the fed group was approximately double that of the unfed group.

**Figure 3:**
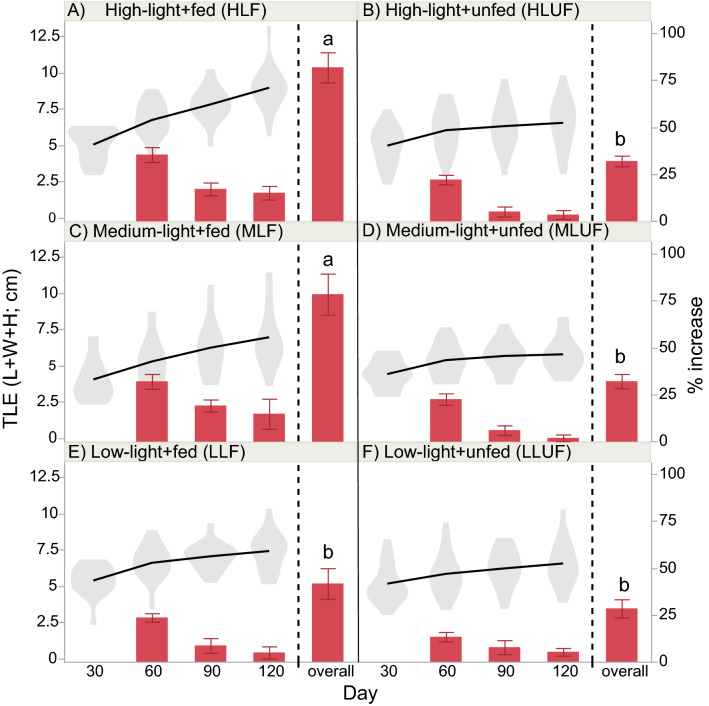
Total linear extension (TLE). TLE (maximum nubbin length + width + height; cm) has been plotted as raw data (violin plots connected by solid lines; left y-axis) or as a % increase (columns & right y-axis). Please note that the rate of increase was calculated against the preceding sampling time only (e.g., the day-90 value represents the % increase since day 60, not the cumulative % increase since day 30.) except in the case of the “overall” column, in which the global ([day 120 − day 30]/day 30*100) % increase was instead computed. Lowercase letters above the overall % increase reflect post-hoc differences across the six light × feeding regime interaction groups. Please note that, although this interaction effect was not statistically significant in the full, mixed-model, repeated measures ANOVA (Table 2), a less conservative, one-way ANOVA across the six groups (box–cox-transformed data) revealed significant differences (*F* = 13.4, *p* < 0.001). Error bars represent standard error of the mean.

The total linear extension (TLE) data (Fig. 3) essentially mimicked these trends since there was a statistically significant (*p* < 0.001), positive correlation (R^2^ = 0.35) between TLE and SGR (evident from the PCA in Fig. 6). Fed corals extended faster than unfed ones (Table 2). HLF (Fig. 3A) and MLF (Fig. 3C) corals displayed the highest rates of increase (~ 80%), with the unfed corals generally displaying rates closer to 30%. The mean TLE and the linear length extension rate in the HLF treatment increased from 5 to 9 cm and from 2 to 3.5 cm over 90 days, which equates to 16 cm and 6 cm year^−1^, respectively.

### F_v_/F_m_ and color

**Figure 4:**
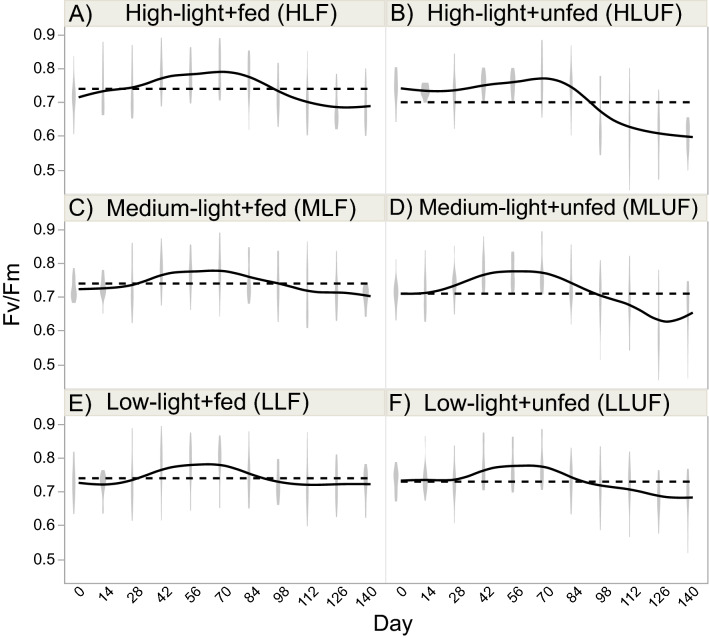
Fv/Fm. Maximum dark-adapted yield of photosystem II (Fv/Fm) was assessed in the 90 nubbins (violin plots connected by solid lines), and the hatched horizontal line reflects the global mean for each treatment. Please note that, unlike for the prior figures, individual mean differences across the six interaction groups have not been shown; although there were numerous such differences, the range from lowest (0.70) to highest (0.74) mean F_v_/F_m_ was so small that it was unclear whether the statistically significant differences were of any biological relevance. Please also note the highly significant tank effect (Table 2).

Feeding regime affected F_v_/F_m_ (Fig. 4; fed > unfed), but, because there was a statistically significant tank effect (Table 2), as well as the actual inter-treatment differences being so small (minimum mean = 0.70 for HLUF and maximum mean = 0.74 for low-light + fed [LLF]), we have not discussed these data at great length. The gradual drop in F_v_/F_m_ for the unfed corals (Fig. 4B,D,F) is worth noting, and the mean decrease from 0.74 to 0.60 in the HLUF group was statistically significant (Tukey’s *p* < 0.05 for day-140 vs. day-0).

**Figure 5:**
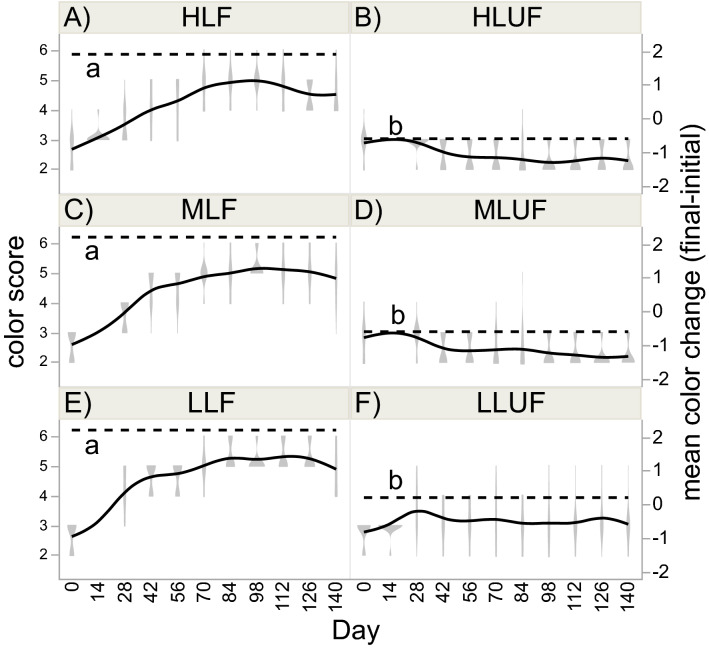
Color scores. Color was assessed in the 90 nubbins (violin plots connected by solid line; left y-axis), and the hatched horizontal line reflects the global mean color score change (final [day-140]–initial [day-0]) for each treatment (right y-axis). Lowercase letters abutting the hatched horizontal lines reflect post-hoc differences across the six light × feeding regime interaction groups. Please note that, because there was no variation across nubbins for certain interaction groups at the 14-day assessment time, the violin plots do not appear (though the solid lines pass through the approximates mean values).

Unlike the aforementioned response variables, both light and feeding regime significantly affected fragment color (Table 2 and Fig. 5); low-light corals presented darker pigmentation. Furthermore, fed corals increased in color over the duration of the study (~ 3 to ~ 5), whereas unfed corals did not (note that the left and right y-axes of Fig. 5 represent raw color scores and changes in color scores, respectively). In fact, the mean color score change of the unfed group (− 0.33) was significantly less than 0 (signed-rank test, *p* < 0.01).

### Multivariate effects

**Figure 6:**
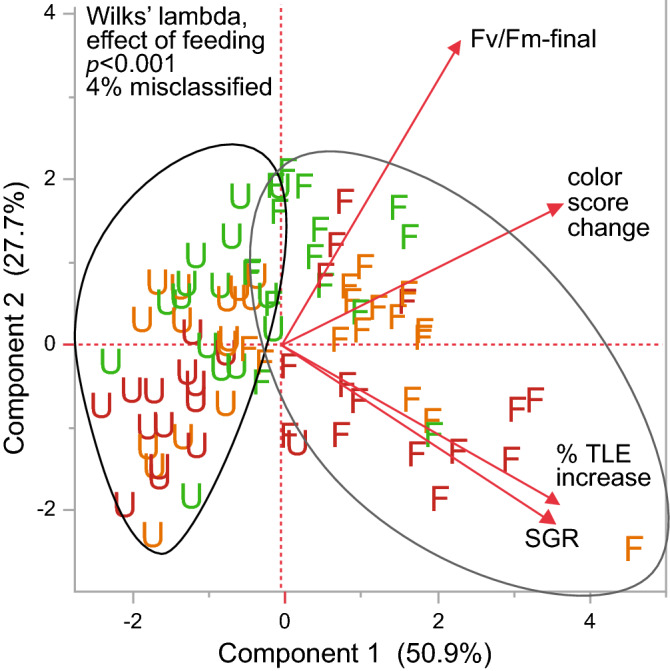
PCA. PCA (on correlations) was carried out on 90 coral fragments for four response variables: the specific growth rate (SGR; based on final and initial buoyant weights only), % increase in total linear extension (TLE), the final F_v_/F_m_ values (day-140), and the color score change (day-140 minus day-0). The results of a MANOVA vs. feeding regime (fed = “F” icons & unfed = “U” icons) have been shown in the upper left corner of the plot. A k-means clustering analysis revealed that 96% of samples clustered with the correct feeding regime group based on the underlying MANOVA model (i.e., 4% misclassification rate). Green, orange, and red icons correspond to low, medium, and high light levels, respectively.

A PCA (Fig. 6) featuring the SGR, color score change, %TLE increase, and final F_v_/F_m_ values captured nearly 80% of the variation in the dataset in the first two PC axes, and a MANOVA of the feeding regime effect on standardized response variable data was statistically significant and revealed that all but 4% of the samples were properly classified into their respective feeding regime groups. In contrast, although the MANOVAs of light and light × feeding regimes were also statistically significant (Wilks lambdas = 0.68 & 0.15, respectively, both *p* < 0.01), the misclassification rates were 42 and 47%, respectively.

## Discussion

Pocilloporid corals were successfully cultured herein for 140 days at a 100% survival rate, and fragments of the HLF treatment quadrupled in size (equivalent to ~ 175 mg g^−1^ week^−1^). This is a significant improvement on the work of Osinga et al.^3^, whose *Pocillopora damicornis* fragments doubled in size (~ 5 to ~ 10 g) over 110 days when fed a daily batch of (1) freshly hatched *Artemia* (starting concentration = 2,000 nauplii cm^−3^) and (2) *Tetraselmis suecica* (starting concentration = 30,000 cells cm^−3^). Osinga et al.^4^ reported BW increases from ~ 0.3 (final concentration of *Artemia* nauplii in tank post-feeding = 0 L^−1^) to ~ 0.7 g (2000–8000 L^−1^) in another *P. damicornis* feeding study. Cunning et al.^36^, in contrast, documented a lower growth rate of cultivated *P. damicornis* specimens without feeding: ~ 43 mg g^−1^ week^−1^. For cultivated *P. acuta* nubbins (6-cm maximum lengths), Conlan et al.^19^ documented highest growth rates (33% BW increase) in those fed *Artemia* nauplii (hatched daily; 0.05 g dry weight tank^−1^ [49 L]) for 90 days.

Field growth rates of pocilloporid corals have been estimated to be lower than the ~ 6 cm year^−1^ measured herein; juvenile *P. damicornis* specimens from nearby our study site grew only ~ 2 cm year^−1^^35^, similar to adult *P. damicornis* growth rates documented at Lizard Island, Australia (2.2 cm year^−1^ for colonies 8–20 cm in diameter^37^) and much higher than those in the Tropical Eastern Pacific (TEP; ~ 0.5 cm yr^−1^ ^38^). Elsewhere in the TEP, Tortolero-Langarica et al.^39^ reported the extension rate of *P. damicornis* to range from 2.24 to 4.55 cm yr^−1^, with Richmond^40^ documenting a wider range of 3.6–6 cm year^−1^ (vs. ~ 1.5 cm year^−1^ in Hawaii in the same study).

The high coral growth rate in the HLF treatment may be due to favorable abiotic conditions, in addition to the high quality and quantity of food. First, the seawater quality in the tanks was suitable for reef coral growth because the chemical dosing system maintained the concentrations of Ca^2+^ and Mg^2+^ at levels recommended by Bartlett^31^. Second, nutrient levels were below detection on account of conducting feeding in a separate tank (sensu Chang et al.^32^), thereby avoiding the eutrophication issue associated with feeding raised by others^31,41^. Third, the addition of live rocks and live sand in RAS improves nutrient cycling by shifting aquarium communities towards more typical seawater assemblages of microbial taxa^11,12^, and this may have directly or indirectly benefited the cultured corals. Finally, the alternation between low and high flow velocity may also have contributed to the fast coral growth rates documented. Reef coral growth, and, more generally, physiology is flow-dependent^42,43^ since seawater flow promotes material exchange and metabolism. For instance, Schutter et al.^42,43^ found a significant interaction between irradiance and water flow on the growth rate of the stony coral *Galaxea fascicularis*. Regardless of light and flow effects, the observation that all cultured corals survived and grew significantly could simply be due to their not being exposed to stressors they would encounter in situ, such as predation, bioerosion, fragmentation (e.g., storm damage), competition, pathogen infection, eutrophication, and/or sedimentation^6,19^.

Corals fed enriched *Artemia* were characterized by faster growth rates, higher Fv/Fm, and darker pigmentation compared to unfed corals, as also documented by other studies^44–47^. Although not measured herein, both symbiont density and chlorophyll concentration increase in fed pocilloporids^45^. *Pocillopora* sp. have shown significantly higher capture rates of *Artemia* nauplii compared to *Acropora* sp.^45,46^, and they were clearly able to capture nauplii herein. Tagliafico et al.^27^ found that coral species fed a lipid-enriched diet (*Artemia* enriched with omega-3 polyunsaturated fatty acids [PUFAs]) grew faster and underwent increases in pigmentation, chlorophyll, and endosymbiont density. These corals also better resisted bleaching. Although bleaching resilience was not tested herein, it is likely that better nourished corals will outperform those relying on autotrophy alone^28^. *Artemia* are cheap, easy to maintain, and could promote cultured coral growth for both restoration efforts and the aquarium trade^1^. This is even more likely to be true given that Lim et al.^47^ found that endosymbionts also benefited when residing within fed, PUFA-enriched corals; this suggests that the holobiont’s capacity for autotrophy could actually be promoted by elevated levels of heterotrophy. Perhaps, then, feeding of *Artemia* would not only benefit industry and restoration, but it could also be used to enhance reproductive output^20^ and improve resilience or even convalesce sick corals^2,48^.

Fed corals increased in color, whereas unfed corals did not. This indicates that feeding, and likely the acquired nitrogen in particular, drove increases in symbiont density and/or chlorophyll concentration, as has been documented by others^16,22,45^. Furthermore, low light-unfed corals presented darker pigmentation than high light-unfed ones, which is also consistent with other reports^16^. In fact, with the exception of color, there were no interaction effects of feeding and light intensity for any response variable; this finding is in agreement with Ferrier-Pagès et al.^14^. Instead, the effect of feeding was far stronger. Houlbrèque and Ferrier-Pagès^30^ suggested that the balance between autotrophy and heterotrophy is dependent on light and other environmental parameters, and the autotrophy vs. heterotrophy shifts in response to increased turbidity^49^ and temperature^25^ in bleached corals support this view. Episodic or ephemeral dependency on heterotrophy during stress events aside, Goldberg^50^ found that some organic constituents of coral tissue are almost exclusively of nonphototrophic origin. Feeding not only provides alternative sources of carbon, nitrogen, and phosphorous, but it also allows for the incorporation of some essential organic constituents (e.g., essential amino acids) that cannot be obtained in sufficient quantities (or at all) via photosynthesis^48^.

Fox et al.^51^ demonstrated that in regions with relatively high primary production (as gauged by chlorophyll-a levels), corals are consistently more heterotrophic. The highest chlorophyll-a values reach 0.8 mg m^−3^, with the monthly average between 0.17 and 0.37 mg m^−3^ (annual mean = 0.25 mg m^−3^) in Nanwan Bay^52^. Such high variation is due to upwelling of cold seawater and nutrients, the latter of which stimulate phytoplankton growth and thereby leads to increased chlorophyll-a concentrations at our study site^53^. Southern Taiwan’s chlorophyll-a concentrations are higher than those of most of the islands mentioned by Fox et al.^51^, thereby potentially signifying that these corals have access to a rich phytoplankton food supply in situ.

In summary, we examined the effect of heterotrophic feeding and light intensity on the physiological performance of the coral *P. acuta*. All corals survived, and fed corals grew at high rates, perhaps not only due to the plentiful food supply, but also to their not being exposed to stressors they would encounter in situ, such as predation, competition, pathogens, eutrophication, and sedimentation^19,54^. It is recommended that those looking to culture stony corals like *P. acuta* in RAS feed them with *Artemia* in independent feeding tanks since this prevented biofouling. The system used herein could be scaled up to mass produce corals for the aquarium trade and provide a sustainable stock for reef rehabilitation efforts and scientific research^55^.

## Methods

### Biological material

Six *P. acuta* colonies (diameter = 12–15 cm) were collected under Kenting National Park permit 1,570,001,572 (to TYF) at depths of 3–5 m in a healthy reef outside the inlet of Taiwan’s third nuclear power plant (21°57′15.7ʺ N, 120°45′21.2ʺ E) in Nanwan Bay, Southern Taiwan in January 2019. Colonies, which were at least 4–5 m apart, were quarantined in the husbandry facility of the National Museum of Marine Biology and Aquarium for two weeks and acclimated in a roofed, outdoor 30-ton flow-through tank characterized by the following conditions: natural seawater filtered to 50 μm, temperature = 26 ± 1 °C (mean ± standard error for this and all other error terms unless stated otherwise), salinity = 35 ± 1, and PAR = 300 μmol quanta m^−2^ s^−1^. After this initial acclimation period, a sterilized scalpel was used to cut fragments to approximately 2 ± 1 cm in length. Each of the six parent colonies generated 15 fragments, and instant glue (Ista, USA) was used to attach them to 2.7-cm, etched, T-shaped ceramic pedestals (Oceanexus, Taiwan; Fig. 1). The 90 fragments were placed in the 30-ton tank under the same conditions as above for recovery and attachment for four weeks, and all survived the preparatory processes.

### Culture systems

The indoor RAS included synthetic seawater: Red Sea salt (Red Sea, USA) mixed with reverse osmosis (RO) water. Each of the three culture tanks included an upper culture tank (125 × 60 × 70 cm) connected to a lower “life support” tank (80 × 45 × 45 cm). The culture tanks featured live rocks (25 kg). LED lights (HLG-480H-C2100B, Taiwan) were positioned above each of the three culture tanks. Corals were placed at ~ 10, ~ 20, or ~ 30 cm depth where they received a constant light level of 250 ± 3.5 (high), 157 ± 8.3 (medium), or 105 ± 5.1 (low) μmol quanta m^−2^ s^−1^ across a 12-h/12-h light/dark cycle, respectively. Each experimental tank also contained two flow motors (Maxspect, GP-03, China), and corals were exposed to alternating low (3.76 ± 0.15 cm s^−1^, n = 10 measurements) and high (6.24 ± 0.23 cm s^−1^, n = 10) flow rates (each for 6 h) to simulate the tidal flow over the duration of the study. It was hypothesized that doing so would better mimic the ebb and slack tides in situ^32^. Light intensity and flow velocity were measured by Li-Cor LI-193SA (USA) and Kenek GR20/GR3T-2-20N (South Korea) meters, respectively.

The life support tank contained a 0.2-mm filter bag, a protein skimmer (CO-2, JNS Aquaria, Taiwan), live sand (3 kg, collected from Nanwan Bay), an automatic Mato-2009 RO bucket (Autoaqua, Taiwan), a zeolite drum (JNS Aquaria, DC-2), a primary pump (Mr. Aqua, 6000 L/H, Taiwan), a titration system (Johnlen, CS072A-1, Taiwan; for measuring alkalinity [as KH], and concentrations of Ca^2+^ & Mg^2+^), a heater (Ista, 350 W), and a chiller (Resun, C-1000 p, China; 26 ± 1 °C). The salinity was maintained at 35 using a Mato-200 osmoregulator (Autoaqua) that automatically compensated for evaporative water loss by periodically adding fresh RO water. Commercial nitrifying bacteria (NBL, A-5 Pandora, Taiwan) were added to the live sand in the bottom of the tanks monthly. In order to ensure consistent water quality and conditioning, the three experimental tanks were connected to one another and operated in a synchronized manner for eight weeks before the experiment was initiated. They were then physically separated when the experiment began.

### Light & feeding treatments

At the beginning of the experiment, 10 fragments were randomly placed at each of the three light levels mentioned above in each of the three culture tanks (n = 30 fragments/tank), where they were cultured for 140 days. Half of the corals were fed with enriched 2-day-old *Artemia salina*, which were prepared as follows. Two days before commencing feeding, 10 g of *A. salina* cysts (Supreme plus, Golden West Artemia, USA) were incubated in a 2-L, well-aerated hatching cone using synthetic seawater for 48 h at 27 ± 1 °C and a salinity of 35 ± 1. The nauplii were enriched by adding 1.5 ml of 100 ppm Pack Boost Enrichment Diets (Omega, Chuan Kuan Enterprise, Taiwan) 36 and 42 h after hatching. A magnetized cyst collector tube was used to remove the unhatched cysts or shells (sensu Tagliafico et al.^27,56^). The 2-d-old *Artemia* nauplii were collected through a 200-μm filter, rinsed with synthetic seawater, and added to an independent tank system that featured an upper feeding tank (120 × 60 × 60 cm) connected to a lower life support tank (80 × 45 × 45 cm). Half of the corals in each tank × light level were fed in this feeding tank, which featured bubble stones in the four corners (to allow for even water mixing). Corals to be fed were nested within a 20 × 55 × 70 cm plankton net (housed within a PVC frame) placed within the feeding tank to ensure that *Artemia* maintained in relative proximity of the nubbins). The *Artemia* density was 42.7 ± 1.75 ind ml^−1^ based on (1) the recommendations of Tagliafico et al.^56^ and (2) the relatively low growth rates observed by Osinga et al.^3,4^ and Toh et al.^9^ when feeding at concentrations of only 2–10 *Artemia* ml^−1^ (see “Discussion”). After the lights were turned off for 30 min, all coral fragments of the fed group were moved into the feeding tank for 4 h, then rinsed with seawater and returned to their respective positions in the experimental tanks. They were fed three times a week.

There were five fragments of each feeding group (fed or unfed) at each of the three light levels in each of the three culture tanks (n = 3 biological replicates; 90 analyzed nubbins in total). Partially synthetic seawater was changed biweekly (30 and 100 L for the culture and feeding tanks, respectively), and concentrations of nitrate, nitrite, phosphate, ammonia, calcium (Ca^2+^), and magnesium (Mg^2+^), as well as carbonate hardness/alkalinity (KH) and pH, were measured biweekly (Salifert Profi Test, Holland).

### Buoyant weight (BW), specific growth rate (SGR), & total linear extension (TLE)

The weights of the coral fragments were measured by a BW technique on a Mettler Toledo AB204 balance (precision = 0.0001 g; USA). A glass beaker containing filtered seawater (26 ± 0.5 °C and a salinity of 35) and a thermostatic bath were placed under the balance, and the coral fragments were suspended on fishing line within the temperature-controlled beakers for BW measurements. Before each measurement, the surface of the coral pedestal was lightly brushed with a toothbrush to remove algae. The SGR (% day^−1^) was calculated as: (ln(W_f_) − ln(W_i_))/Δt × 100), where ln(W_i_) and ln(W_f_) represented the natural logarithms of the coral fragment BW (g) at the beginning and the end of the experiment, respectively, and ∆t represented the duration in days. Vernier calipers were used to measure the lengths, widths, and heights of the fragments, and TLE (sensu Kikuzawa et al.^57^) was calculated as length + width + height. TLE data were analyzed as raw data (cm) and as % increase.

### Maximum quantum yield & color

The maximum, dark-adapted photosynthetic yield of photosystem II (F_v_/F_m_) was measured biweekly using pulse amplitude modulation (PAM) fluorometry (settings: saturation pulse intensity = 11, measurement light intensity = 11, gain = 8, damp = 2; Diving PAM, Walz, Germany). After turning off the light for 30 min, both minimum (F_o_) and maximum fluorescence (F_m_) were measured for each fragment, and F_v_/F_m_ was calculated as F_v_/F_m_ = (F_m_-F_o_)/F_m_. Coral fragments were photographed with a fixed light source (5500 K, LED) in a 40 × 40 × 40 cm studio biweekly using a TG5 digital camera (Olympus). Based on CoralWatch’s “Coral Health Chart”^58^, fragments were scored along the D1 to D6 axis, and the color scores were assessed as raw score data or as the difference of the final and initial score.

### Statistical analyses

Data were tested for normality (Shapiro–Wilks test) and equal variance (Levene’s test), and box-cox transformations were generally required for most response variables. When box-cox transformation did not yield normally distributed residuals, rank-transformations followed by non-parametric analyses were instead conducted. Four different statistical models were used. First, a mixed-model RM ANOVA was used with fragment nested within the intercept as a random effect; this was done to ensure that the response variables did not vary significantly across fragments at time 0 (none did; *p* > 0.05 for all). For the raw BW, raw TLE, Fv/Fm, color score, and seawater quality data, 2-way RM ANOVAs were then used to test for the effects of light (3 levels), feeding regime (fed vs. unfed), and their interaction over time with JMP Pro (ver. 14); sampling time and coral fragment were the RM and repeated-subject, respectively, and tank was nested within the light × feeding regime interaction (main, fixed effects), as well as within light level to accommodate the split-plot nature of the experiment. JMP Pro’s “unequal variances” RM ANOVA mixed-model type was used because it permits the inclusion of both random factors (the split plot) and RM. For the response variables assessed at the final sampling time (day-140) only-SGR, TLE % increase, and color change- a simpler, ANOVA was conducted with light, feeding regime, their interaction, and tank as the fixed factors; tank was also nested within light level as a random factor (split plot), and colony of fragment origin was considered as a secondary random element. Finally, a simpler, less conservative 1-way ANOVA was carried out with the six interaction groups (light × feeding regime) as the fixed factor since such a test best reflects what is depicted in the manuscript’s figures. For all ANOVA models, an alpha level of 0.01 was set, and Tukey’s and Dunn’s multiple comparisons were used to detect individual mean differences for the parametric and nonparametric analyses, respectively (alpha = 0.05). Only untransformed means were plotted. Principal components analysis (PCA; on correlations) and multivariate ANOVA (MANOVA; standardized data) were used to depict multivariate effects of light and feeding regime with the final sampling time data (SGR, % TLE increase, final F_V_/F_M_, and color change), and k-means clustering was used to cluster samples by feeding regime. All statistical analyses were carried out by JMP Pro.
